# Making the Ask: Best Strategies for Advocacy and Appropriation Requests

**DOI:** 10.1093/geroni/igaf122.617

**Published:** 2025-12-31

**Authors:** Tracy Fasolino

**Affiliations:** Clemson University, Clemson, South Carolina, United States

## Abstract

‘Waste, fraud, and abuse’ are the main narratives ringing throughout the 119th congressional corridors, regardless of political affiliation or party alignment. Research, education, and public health are posed for significant revisions in staffing and services, and the consequences will likely be felt for years. The traditional strategies for seeking federal funding and support will fail to produce the anticipated outcome given this season of governmental cuts. New approaches for ‘making the ask’ on behalf of advocacy groups and during appropriation season will be necessary. As a Health & Aging Policy Fellow Residential Track with Senator Tim Kaine (D-VA), Dr. Tracy Fasolino witnessed first-hand the rapid changes during these unprecedented times with the flurry of Executive Orders and the dismantling of many executive branch organizations. She gained valuable insight into the best strategies for engaging congressional staff while advocating for a particular cause or initiative. During appropriation season, she developed a keen understanding of the process and can offer some insights on how to reframe the narrative. She will share new approaches for presenting requests in a bipartisan and bicameral approach beyond the typical data analytics and storyboarding. As the new administration continues with budgetary cuts, fresh approaches and restructured narratives will be necessary.

